# Extreme events are more likely to affect the breeding success of lesser kestrels than average climate change

**DOI:** 10.1038/s41598-020-64087-0

**Published:** 2020-04-29

**Authors:** J. Marcelino, J. P. Silva, J. Gameiro, A. Silva, F. C. Rego, F. Moreira, I. Catry

**Affiliations:** 1 Centre for Applied Ecology “Prof. Baeta Neves”/InBIO Associate Laboratory, Instituto Superior de Agronomia, Universidade de Lisboa, Tapada da Ajuda, 1349-017 Lisboa, Portugal; 2 CIBIO/InBIO, Centro de Investigação em Biodiversidade e Recursos Genéticos, Laboratório Associado, Universidade do Porto, Campus Agrário de Vairão, 4485–601 Vairão, Portugal; 3 CIBIO/InBIO, Centro de Investigação em Biodiversidade e Recursos Genéticos, Instituto Superior de Agronomia, Laboratório Associado, Universidade de Lisboa, Tapada da Ajuda, 1349-017 Lisboa, Portugal; 4 REN Biodiversity Chair, CIBIO/InBIO-UP, Centro de Investigação em Biodiversidade e Recursos Genéticos, Universidade do Porto, Campus Agrário de Vairão, Rua Padre Armando Quintas, 4485–601 Vairão, Portugal; 5 cE3c, Centre for Ecology, Evolution and Environmental Changes, Faculdade de Ciências da Universidade de Lisboa, Campo Grande, 1749-016 Lisboa, Portugal; 6 Instituto Português do Mar e da Atmosfera, I.P., Rua C do Aeroporto, 1749-077 Lisboa, Portugal

**Keywords:** Grassland ecology, Climate-change impacts, Projection and prediction, Climate-change ecology

## Abstract

Climate change is predicted to severely impact interactions between prey, predators and habitats. In Southern Europe, within the Mediterranean climate, herbaceous vegetation achieves its maximum growth in middle spring followed by a three-month dry summer, limiting prey availability for insectivorous birds. Lesser kestrels (*Falco naumanni*) breed in a time-window that matches the nestling-rearing period with the peak abundance of grasshoppers and forecasted climate change may impact reproductive success through changes in prey availability and abundance. We used Normalised Difference Vegetation Index (NDVI) as a surrogate of habitat quality and prey availability to investigate the impacts of forecasted climate change and extreme climatic events on lesser kestrel breeding performance. First, using 14 years of data from 15 colonies in Southwestern Iberia, we linked fledging success and climatic variables with NDVI, and secondly, based on these relationships and according to climatic scenarios for 2050 and 2070, forecasted NDVI and fledging success. Finally, we evaluated how fledging success was influenced by drought events since 2004. Despite predicting a decrease in vegetation greenness in lesser kestrel foraging areas during spring, we found no impacts of predicted gradual rise in temperature and decline in precipitation on their fledging success. Notwithstanding, we found a decrease of 12% in offspring survival associated with drought events, suggesting that a higher frequency of droughts might, in the future, jeopardize the recent recovery of the European population. Here, we show that extreme events, such as droughts, can have more significant impacts on species than gradual climatic changes, especially in regions like the Mediterranean Basin, a biodiversity and climate change hotspot.

## Introduction

Climate change is an unequivocal anthropogenic induced threat with clear and widespread impacts on natural systems, predicted to surpass habitat destruction as the greatest global menace to biodiversity^1^. Warmer temperatures and altered precipitation regimes are already affecting biological diversity through effects on ecosystems, species and ecological interactions^2–4^. These alterations are likely to negatively affect not only the habitat quality of many animal species but also their behaviour, distribution and breeding phenology^5–8^. Although birds’ high mobility makes them highly reactive to changes in their environment, it is essential to evaluate their plasticity and how climate change can impact them, in order to improve assessments of risk and develop adaptation strategies.

For many bird species, the impacts of climate change are likely to operate indirectly, through alterations in both food abundance and availability^9^. In fact, warmer temperatures and droughts affect plant phenology and consequently insect emergence, which cause many birds to adjust their breeding strategy^10^. These widespread phenological changes can result in desynchronization between trophic availability and peak demand of resources during key biological stages such as breeding^11,12^, and consequently contribute to a decrease in their breeding success, which ultimately can lead to population decline^13^.

Beyond gradual changes in temperature and precipitation, one of the most harmful consequences of climate change for birds may be the increasing frequency, intensity and duration of extreme climatic events (herein, “extreme events”), such as heatwaves and droughts^14–17^. Recent studies have described lethal and sublethal fitness costs for different species including decreases in body condition in songbirds when exposed to record low temperatures^18^; massive reproductive failure when exposed to unusual warm summers^19^; deaths of thousands of individuals when exposed to severe heatwaves^20^, among other costs^21–24^. In addition to exceeding the physiological tolerances of several species, extreme events can have more significant repercussions on population persistence than gradual climatic changes^25,26^ and currently face outstanding challenges in their prediction^16,27^, as they depend on stochastic processes. Furthermore, current model evaluation tools are not specifically suited for the analysis of extremes^28^.

The Mediterranean region is a climate change^29^ and a biodiversity^30^ hotspot, making this region one of the most vulnerable regions in the world^31,32^. While global average annual temperature has risen by 1°C since the end of the XIX century, in the Mediterranean region, the temperature is now 1.4 degrees higher for the same period^33^. Future projections predict a regional rise in temperature from 2 to 6°C depending on the climate scenario and season^34^. Additionally, these long-term trends also show a decrease in precipitation (−4% to −27%) and an increase in drought periods with more frequent and extreme heatwaves^35–37^.

Here, we address the impacts of forecasted climate change on the lesser kestrel (*Falco naumanni*), in their Mediterranean breeding grounds. Mediterranean cereal steppes are one of the most valuable habitats in Europe, due to their aesthetic, cultural and ecological value^38,39^. Although steppe birds are adapted to periods of low food availability in the dry season^40^, current climate conditions could surpass their resilience capacity^41^. In fact, impacts on grassland ecosystems will not only change the vegetation structure and biomass, two crucial determinants of habitat quality for steppe birds^42,43^ but will also likely impact the availability of trophic resources, influencing birds’ behaviour, distribution and breeding success, constituting an additional threat to many endangered species^7,44,45^. Whilst there is virtually no information on the impacts of climate change on steppe birds, this information is vital for adaptive management.

Satellite-derived Normalized Vegetation Index (NDVI) is a measure of vegetation greenness and biomass, used to distinguish patterns of vegetation productivity and also one of the most used tools to monitor biodiversity at large spatial and temporal scales^46^. As it correlates with vegetation productivity, it has been used as a surrogate for habitat quality and to describe bird distribution and performance^47–50^. Vegetation greenness is linked with temperature and precipitation^51,52^ and with primary consumers abundance like insects and small mammals^53–56^. In this study, we explored the use of NDVI as a surrogate of habitat quality (i.e. higher vegetation biomass expected to indicate higher prey abundance) to assess how climate change will impact the breeding performance of lesser kestrels, through changes in food availability. The lesser kestrel is a colonial migratory raptor inhabiting steppe-like habitats in southern Europe that has undergone a great decline in the nineties, recovering after several interventions at the beginning of the twenty-first century. Lesser kestrel feeds mainly on Orthoptera grasshoppers, locusts and crickets, and large beetles^57–60^. Foraging areas include open agricultural landscapes with little ligneous vegetation like grasslands, steppe-like habitats, pastures and non-intensive cultivations^61^. Food availability is of extreme importance for their breeding success: females with better body condition lay earlier, produce larger clutches and more offspring^43,62^, and limited foraging availability influences nestling body condition and annual fledging success^58,63,64^.

Using breeding data from a long-term study (2004–2017) on the lesser kestrel population in southern Portugal we focused on two main goals: (1) predict future NDVI and fledging success according to IPCC Fifth Assessment’s future climate change scenarios and (2) evaluate how fledging success is influenced by extreme drought events.

To answer our first objective, we tested whether spring NDVI around lesser kestrel colonies could predict fledging success. Next, we used current climatic variables to explain spatial (different colonies) and temporal (annual) variation in the NDVI. Then, based on the relationship between NDVI and climate variables, we used future climatic scenarios to estimate NDVI spring values for 2050 and 2070 and use this information to predict how fledging success may be affected by future climatic conditions. Finally, we analysed how drought events between 2004 and 2017 affected fledging success to further investigate the potential impacts of the increasing frequency of extreme events on lesser kestrel breeding performance.

## Results

### Lesser kestrel fledging success and NDVI

**Figure 1:**
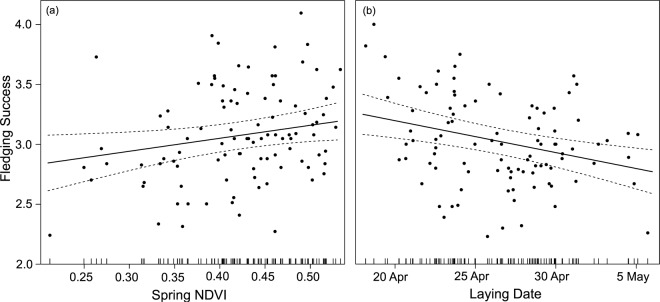
Effects of (**a**) spring satellite-derived Normalized Difference Vegetation Index (NDVI) and (**b**) laying date on annual lesser kestrel fledging success between 2004 and 2017. Each dot corresponds to one colony in a given year (n = 100 colony-years). Rug plots in the x-axis represent the distribution of the observed values.

Fledging success values varied between 2.23 and 4 (mean = 3.03 ± 0.38). Annual spring NDVI values varied between 0.21 and 0.53 (mean = 0.42 ± 0.07) and the laying date ranged between 108.2 (18 April) and 125.7 (5 May) julian days (mean = 116.4 ± 4.09). Mean spring NDVI around lesser kestrel colonies positively affected fledging success (β = 1.03 ± 0.46, p = 0.026, Fig. 1a), suggesting mean spring NDVI to be a reliable predictor of fledging success. Colonies surrounded by foraging patches with greener vegetation produced more fledglings. Lesser kestrel fledging success was negatively correlated with laying date (β = −0.027 ± 0.008, p < 0.0001, Fig. 1b), with colonies showing earlier laying dates producing more fledglings.

### NDVI and aridity index

**Figure 2:**
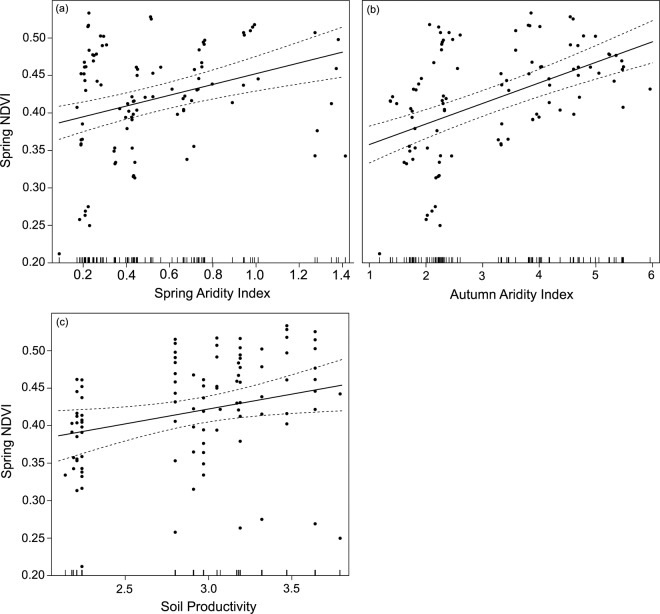
Effects of (**a**) autumn aridity index, (**b**) spring aridity index and (**c**) soil productivity on spring satellite-derived Normalized Difference Vegetation Index (NDVI) around lesser kestrel colonies between 2004 and 2017. Rug plots in the x-axis represent the distribution of measured values.

Spring and autumn aridity indexes around lesser kestrel colonies from 2004 to 2017 varied between 0.09 and 1.41 (mean = 0.52 ± 0.32), and between 1.1 and 6.5 (mean = 3.20 ± 1.32), respectively. Soil productivity ranged from 2.14 to 3.79 (mean = 2.90 ± 0.49). Winter aridity index variable did not influence NDVI (p = 0.513). Mean spring NDVI around colonies was positively influenced by autumn (β = 0.03 ± 0.004, p < 0.0001) and spring aridity index (β = 0.07 ± 0.02, p < 0.0001), and soil productivity (β = 0.04 ± 0.02, p = 0.022). Both spring and autumn aridity indexes showed similar positive patterns, with drier years showing lower spring NDVI values (Fig. 2). Soil productivity also showed a positive correlation – more productive soils were associated with higher NDVI. We did not find evidence of spatial autocorrelation in the residuals of this model (Figure S1, SM).

### Forecasting vegetation greenness (NDVI) and lesser kestrel fledging success

**Figure 3:**
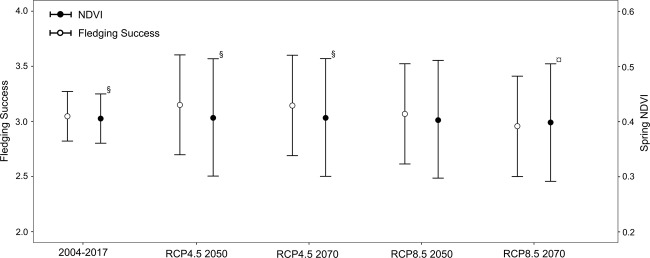
Predicted spring Normalized Difference Vegetation Index (NDVI, filled circles) and fledging success (open circles) with 95% confidence intervals for RCP climatic scenarios 4.5 and 8.5 for years 2050 and 2070. Observed spring NDVI and fledging success values between 2004 and 2017 is shown for comparison. Bars sharing different symbols (superscript) are significantly different (p < 0.05).

NDVI was predicted for models MPI-ESM-LR and HadGEM2-ES, and RCP 4.5 and 8.5 scenarios for 2050 and 2070, using the corresponding expected values of the aridity index. We did not find significant differences in forecasted NDVI values between global climate models for 2050 and 2070. Consequently, data from both GCMs was analysed together (Fig. 3). Forecasted spring NDVI values for RCP4.5 scenario varied between 0.34 and 0.52 (mean = 0.43 ± 0.03), and for RCP8.5 ranged from 0.31 to 0.49 (mean = 0.40 ± 0.03). We found statistically significant differences between mean spring NDVI values under scenario RCP8.5 from 2070 and three other scenarios: present spring NDVI (p = 0.036) and scenarios RCP4.5 for 2050 (p < 0.001) and 2070 (p < 0.001, Fig. 3). The scenario RCP8.5 from 2070 consistently showed lower values relative to the other scenarios and the studied period (2004–2017).

Fledging success was predicted for each climate change scenario based on the forecasted NDVI for the years 2050 and 2070. Predicted fledging success values for the RCP4.5 scenario varied between 2.50 and 3.57 (mean = 3.03 ± 0.15), and for RCP8.5 ranged from 2.46 to 3.55 (mean = 3 ± 0.15). We found no significant differences in mean fledging success between scenarios and years (χ^2^ = 1.205, df = 4, p = 0.877, Fig. 3).

### Extreme events

**Figure 4:**
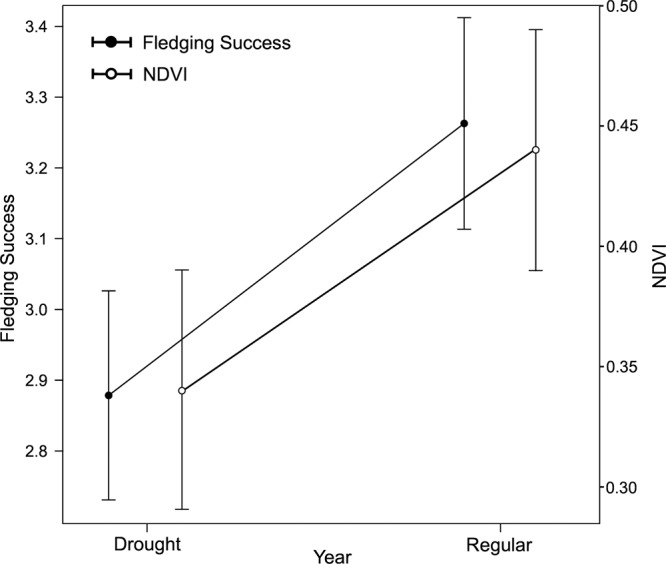
Lesser kestrel fledging success and spring Normalized Difference Vegetation Index (NDVI) for drought (2005, 2009, 2012) and regular (2004, 2007, 2016) years. Bars represent confidence intervals.

The years 2005, 2009 and 2012 were considered to be the three years with more extreme drought months between 2004 and 2017, considering a mean 3-month SPI of −1.00 ± 0.8 (min = −2.43, max = 0.53). In contrast, the years 2004, 2007 and 2016 were the most regular years, with SPI values close to zero (mean = 0.01 ± 0.8, min = −1.06, max = 1.41), without severe droughts. We found significant differences in mean fledging success between regular and extreme drought years (β = 0.29 ± 0.08, p < 0.0001), with significantly lower fledging success in extreme drought years ($\overline{FS}\,$_normal_ = 3.26, $\,\overline{FS}\,$_drought_ = 2.87, Fig. 4).

## Discussion

Many studies have focused on the impacts of mean, long-term changes in climate on biodiversity, disregarding the effects of extreme weather events^5,9,12,65^. Our study shows that, while forecasted mean fledging success was found not to suffer significant changes in the estimated average temperature and precipitation variation for the Mediterranean area, extreme droughts in the last decade were found to be associated with significantly lower fledging success.

NDVI has been used to predict richness, distribution and abundance of birds^66–68^ using vegetation greenness as a proxy of food availability, but not directly relating NDVI to fledging success. Indeed, previous studies have shown positive relationships between NDVI and insect abundance - in recent years, it was used to determine locust and grasshopper abundance and potential habitat availability^69,70^, to determine beetle species richness and abundance^54,71^, as a proxy of grasshopper abundance to track movements of harriers (*Circus pygargus*) during winter^72^, to predict prey abundance for great (*Parus major*) and blue tits (*Cyanistes caeruleus*)^55^ and as a proxy of small mammal abundance, one main prey for American kestrels (*Falco sparverius*)^56^. Because the diet of lesser kestrels’ offspring consists mainly of grasshoppers^60^ that feed on grasses and leaves and are thus dependent on green vegetation, we used primary productivity in spring (quantified by NDVI) as a proxy of grasshopper abundance to show that temporal (annual) and spatial (different colonies) changes in spring NDVI can predict annual fledging success of lesser kestrels. Fledging success was positively associated with spring NDVI, suggesting that higher levels of plant productivity in foraging areas are likely to increase prey abundance. Years and colonies with lower NDVI values, i.e., with less vegetation biomass in spring, were found to have lower fledging success. Earlier laying dates were found to be associated with higher fledging success, validating previous research showing that seasonal declines in breeding performance are a commonly observed pattern in birds^62,73–75^.

Spring and autumn aridity index, integrating precipitation and temperature, successfully predicted temporal and spatial variation in NDVI around lesser kestrel colonies. Soil moisture, which is regulated by precipitation and temperature regimes, influences plant growth^76^ and consequently, the time span of vegetation biomass. Drier and warmer years are characterized by lower vegetation greenness and biomass (lower NDVI) and, possibly, to less available prey in spring, leading to a decline in lesser kestrel fledging success. Soil productivity was also a good predictor of vegetation greenness around kestrel colonies – more productive soils can retain more moisture and have higher vegetation biomass, likely contributing to higher grasshopper abundance.

### Impacts of gradual climate change and extreme climatic events on fledging success

In the future, forecasted climate changes in the region include an increase in temperature and frequency of heatwaves and also more frequent drought periods^35,37^, anticipating a decline in the greenness of fields and shortening the time span of availability of green areas around lesser kestrel colonies.

Our results show that NDVI in spring is likely to decrease under the more extreme climate change scenario for 2070 (RCP 8.5 - expected continuous increase of the greenhouse gas emissions), compared with present and RCP 4.5 (expected stabilization of the greenhouse gas emissions) scenarios, but, contrary to what would be expected, lesser kestrel fledging success was predicted not to vary significantly under any of these scenarios. Considering that each pair needs to raise at least 2.4 fledglings/year to maintain a viable population^43^, and that none of our predictions went below this threshold, it is expected that, even under the more severe climate change scenarios of gradual increase in temperature and decrease in precipitation (RCP 8.5), the viability of the population will not be directly affected through food availability. Nevertheless, we should stress that this study did not consider changes in land use and agricultural management practices and that interaction effects between climate and land use changes should be quantified^77,78^. Moreover, impacts of climate change and extreme events on adult survival were not accounted for, and should also be considered as population viability does not depend solely on the breeding output. Finally, we have to consider that climate projections have limitations when working at a local scale – the predictions are not as accurate as on a global scale and have great difficulty in predicting extreme events^28^, and so our predictions could be underestimating the effects of the gradual climate change effects in food availability for lesser kestrels. Indeed, climatic vulnerability assessments are usually focused on how mean temperature and changes in precipitation regimes will affect species and ecosystems, but fail on predicting the impacts of extreme events given their rarity and stochasticity^79^.

In our 15-year study, the years of 2005, 2009 and 2012 provided a good example of lesser kestrel nestlings responses to extreme drought periods. In years with extreme drought months, the percentage of nestlings that fledged decreased by 12%, when compared to years without such events. Our results show that, although the predicted increase in mean temperatures and a decrease in precipitation caused by climate change is not likely to affect lesser kestrel fledging success in Southwestern Iberia in the near future, the likely increase in extreme climatic events, such as droughts, can significantly impact the productivity of this species through a decline in prey availability. Previous studies^80,81^, reported heat-related die-offs amongst lesser kestrel nestlings due to hyperthermia and acute dehydration; within survivors, heat events significantly increased physiological stress levels and mass loss (up to 30% of body weight) suggesting that lesser kestrel nestlings have relatively low tolerance to high environmental temperatures. In the future, the accumulated impacts of increased frequency of droughts and heatwaves can worsen the lethal and sublethal fitness costs for lesser kestrels, decreasing both survival and fledging success and likely reverting the positive recovery trend of the lesser kestrel populations.

## Conclusions

While many studies have linked NDVI with species richness, distribution and abundance^82^ we did not find studies that directly relate this vegetation index with fledging success. Our study is one of the first successfully making this connection and predicting fledging success under different climate change scenarios. In the future, lesser kestrels fledging success will most likely be affected by extreme droughts than gradual rises in mean temperature. As pointed out before^17^, our results highlight that research on the impacts of climate change should not only focus on how species and ecosystems will respond to gradual long-term changes in climate, but also, and perhaps more importantly, on how changing frequency of events such as droughts and heatwaves will directly or indirectly impact birds. In fact, other bird species have been reported to be impacted by extreme weather events^18,83–89^ highlighting the need for more studies to fully understand how species and populations will be affected in the future by such events.

Whilst the uncertainty on the prediction of extreme events makes conservation measures difficult to implement at a local level, the same actions that promote adaptation to gradual changes in climate should benefit species threatened by extreme events^90^. These efforts are based on enhancing habitat connectivity and access to climate refugia^91–93^ (areas buffered from climate change) for the conservation of intact habitats, and adaptive management for individual sites and populations to increase species resilience to track climate change^9^. Whilst previous studies in our study area identified the modification of nest-site provisioning as an effective conservation action to decrease direct nestling mortality and increase lesser kestrel resilience to high temperatures in a more effective way^80^, recommendations to arrest the indirect impacts of extreme events through decreases in prey availability identified here are more challenging to implement.

Lesser kestrels are highly dependent on suitable foraging habitats and land-use changes have been pointed as one major threat for their populations^94,95^. In Portugal, the Castro Verde SPA holds over 80% of the Portuguese lesser kestrel breeding population with a positive trend, while breeding areas north of it (e.g. SPAs of Cuba, Vila Fernando and Campo Maior) – that can be used as climate refugia – have smaller and declining populations, as a result of inadequate land management measures. Ensuring suitable foraging grounds and halting habitat loss within these areas would be essential to promote access to refugia, enhance habitat connectivity, and consequently to increase lesser kestrel resilience to ongoing climate change. These conservation actions would not only likely benefit lesser kestrels but also many other grassland bird species that depend on this habitat and are threatened by climate change.

## Materials and Methods

### Study area and species

The study was conducted in the Castro Verde Special Protection Area (SPA, 37°44′43.0′′N 8°00′32.0′′W), the main area of cereal steppe landscape in Portugal^96^ created for steppe bird conservation under the European Birds Directive and comprising 80% of the national lesser kestrel breeding population, estimated at 557–622 breeding pairs^97^. The area is characterized by a typical Mediterranean climate, with hot, dry summers, reasonably cold winters and low annual rainfall^98^. The traditional agricultural system in the region is based on rain-fed cereal cultivation using a rotational scheme with fallow fields. Cereal is cultivated for two consecutive years, and then it is left fallow for 3–5 years. In recent years, the traditional system has shifted to specialized production of cattle or sheep, leading to an increase of permanent pastures and hay^99,100^.

Lesser kestrels start arriving from their African sub-Saharan wintering grounds in February^101^, occupy cavities in abandoned rural structures or artificial nesting boxes, and lay four to six eggs between April and May. They are colonial breeders, single-brooded, with an incubation period of 28 days, and nestlings fledge 35 to 37 days after the hatching date^102^. Lesser kestrels hunt predominantly in extensive agricultural areas around the colonies, especially in fallows and cereal fields (during harvesting), and avoid scrubland and forests^95,103^.

### Breeding parameters

**Figure 5:**
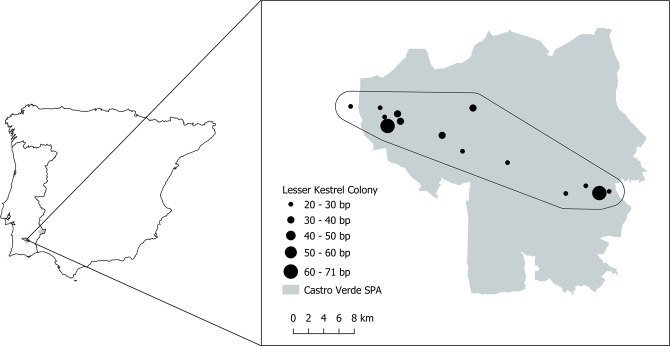
Spatial distribution of lesser kestrel colonies around the Castro Verde SPA. Black circles size represents the mean colony size for the study period. The study area represented by the black line is the convex hull of a 2 km buffer around each colony.

From 2004 to 2017, 15 lesser kestrel colonies (mean colonies/year = 7 ± 1.8, min = 4, max = 10; mean breeding pairs/colony = 31.9 ± 14, Fig. 5) were monitored on a weekly basis over the breeding season (April to July). Since not all colonies were monitored in all years, we only observed a total of 100 colony-year combinations. For each colony and year, mean values for laying date, clutch size and fledging success (n = 100 colony-years) were calculated following the procedures described in Catry *et al*.^62^. Fledging success was quantified as the mean number of nestlings fledged in nests where at least one nestling fledged and was used as a productivity indicator for this species. All colonies, except for one, were located inside the Castro Verde SPA with a maximum and minimum distance between colonies of 35.8 km and 0.14 km, respectively (mean = 15.5 ± 10.1 km).

### NDVI around lesser kestrel colonies

To calculate NDVI around colonies, we defined a 2 km gridded buffer for each colony, as this is the area where most foraging trips take place during the nestling-rearing period^95,103^. To prevent bias in NDVI estimation caused by including unsuitable habitats for lesser kestrels, we used a Corine Land Cover map for 2006^104^ and the farm characterisation from Ribeiro *et al*.^100^, to verify the land uses inside the buffer. The dominant land use around colonies included open habitats such as temporary pastures, followed by permanent pastures and fallows. Unsuitable foraging habitats include forest, orchards and olive, horticultural areas, water and infrastructures (as buildings and roads)^95^; thus, all cells (250 × 250 m) of the grid with less than 95% of suitable open area were excluded from the analysis.

NDVI values around lesser kestrel colonies were estimated using 8-day composite 250 m spatial resolution MODIS (Moderate Resolution Imaging Spectroradiometer) images^105^ – product MOD09Q1 – of the study area (tile horizontal 17, vertical 5) from 2003 to 2017. All images were visually inspected for cloud cover, and cloudy pixels were excluded from the analysis to avoid accounting for areas obscured by clouds. Any images with more than 25% cloud cover across the study area were also discarded, following the procedure of Smith *et al*.^56^. NDVI was calculated as the difference between the near infra-red (NIR) and the red (R) reflectance values over the sum of the two^106^:

1 $$NDVI=\frac{NIR-R}{NIR+R}$$

Annual spring NDVI, the period with the most considerable variation of vegetation greenness and that comprises all the breeding phenology stages of the species (Figure S2, SM), was used to predict annual fledging success. It was computed as the mean NDVI between April and June in all 250 m pixels with over 95% of suitable habitat.

### Climatic predictors of NDVI

To assess the effects of weather conditions on the spatial and temporal variation of satellite-derived NDVI, we modelled the relationship between the seasonal variation of NDVI for the period 2004–2017 around the 15 lesser kestrel colonies and an index of aridity (hereafter aridity index). The aridity index (AI) combines precipitation and temperature – climatic variables with a strong influence on the NDVI^76^. It is calculated as a function of precipitation, potential evapotranspiration and temperature^107^:

2 $$Aridity\,index\,(AI)=\frac{P}{\overline{PET}}$$

where P is the mean precipitation, and $\overline{PET}$ is the potential evapotranspiration in a given period, with higher values of AI indicating more humid conditions and lower values more arid conditions.

Daily rasters of precipitation and maximum and minimum temperatures were obtained from the Portuguese Institute of the Sea and the Atmosphere (IPMA) for the period 2004-2017 at a 1 km resolution (IPMA 2018, unpublished). PET was estimated based on this data using the 1985 Hargreaves evapotranspiration equation^108,109^:

3 $$PET=0.0023\,\times {R}_{a}\times (Tmean+17.8)\times Trang{e}^{0.5}$$

where R_a_ is the radiation on top of atmosphere obtained from CGIAR-CSI^110^, T_mean_ is the mean temperature for the time period, and T_range_ is the time period range of temperatures.

The AI was computed with a 1 km precision for the open area in the 2 km buffer around colonies for spring (April – June), autumn (October – December) and winter (January – March) seasons, as temperature and precipitation in the previous months could influence NDVI values during spring.

We also included soil productivity as a predictor of NDVI to account for spatial variation in soil quality, a factor that was expected to affect NDVI. The soil ecological value^111^, which indicates a scale of the relative importance of soils in Portugal, including their productive and ecological potential, is permanent across years and was used as an effective proxy of soil productivity^112^. Taking into account physical, chemical and biological aspects of soils, it measures the capacity of the soil to sustain biomass.

All NDVI, AI, precipitation and temperature images were analysed, transformed and manipulated using libraries ‘rgdal’, ‘raster’, ‘gdalUtils’ and ‘rgeos’ in R 3.5.1^113^.

### Climate projections

Climatic data projections for 2050 and 2070 were obtained from the Intergovernmental Panel on Climate Change (IPCC) through WorldClim 1.4 database^114^ at 1km^2^ resolution. Future aridity indices were derived from two General Circulation Models (CGMs) from CMIP5 (IPCC Fifth Assessment), - MPI-ESM-LR and HadGEM2-ES - as they are the most suitable projections for the Mediterranean Basin^115,116^ – and Representative Concentration Pathways (RCP) 4.5 and 8.5 scenarios^117^, characterized, respectively, by the stabilization and the continuous increase of the greenhouse gas emissions. The RCP 4.5 scenario represents a moderate warming trend, with a mean global increase of 1.4°C by 2050 and 1.8 by 2070, and the RCP 8.5 represents an extreme warming trend, with an increase of 2°C and 3.7°C, respectively for the same periods^32^.

### Impact of Extreme Events

Although predicting the occurrence of individual extreme events and their inclusion in forecasted scenarios is a great challenge, impacts of climate change should account for changing frequency of extreme climatic events, rather than focusing only on gradual changes in average values. To evaluate how fledging success is influenced by extreme events and predict the impacts of forecasted increases in their frequency, we used past occurrences, choosing the three drier years with at least one month with Standardized Precipitation Index (SPI) values below −1.5 (corresponding to severe and extreme drought^118^ and the three more regular years (monthly values close to zero and no presence of extreme droughts or rains) from 2004 to 2017, according to 3-month SPI values^119^.

## Data analysis

We used satellite-derived NDVI series (2004-2017) to relate climate, habitat quality and lesser kestrel fledging success. First, the hypothesis that NDVI predicts lesser kestrel fledging success was tested using general linear mixed-effects models using 15 colonies and 14 years of data (n = 100 colony-years). The response variable was fledging success, fixed effects were mean spring NDVI and laying date, and a random effect (colony ID) was used to control for the repeated sampling of the kestrel colonies. Secondly, we tested if climate variables were able to predict NDVI, using seasonal AI and soil productivity as fixed effects and kestrel colony as a random variable to control for repeated sampling in the same areas. Model adequacy was evaluated by checking the normal distribution of the residuals and marginal and conditional R^2^. The latter model was used to forecast NDVI values for all climatic projection scenarios and models for 2050 and 2070 based on spring and autumn AI expected values for those years. Using the same procedure, NDVI expected values from the first model were used to predict future fledging success for the same projections. All independent variables were checked for correlations.

Mixed models were fitted using the package ‘lme4’^120^ in R 3.5.1^113^. We computed R^2^ values for mixed models in R^113^ using Nakagawa *et al*.^121^ method in ‘piecewiseSEM’ package^122^. To evaluate predictive model performance, we applied bootstrapping procedures to each model. As predictive metrics, we calculated the Root Mean Square Error (RMSE) for the GLMM using functions subBoot and RMSE.merMod from the package ‘merTools’ in R. Backward elimination of non-significant explanatory variables was used to reach the full models.

To check for differences between the mean fledging success in regular and extreme drought years, we used general linear mixed models using a subset of 6 years of data and 12 colonies (n = 45 colony-years). The response variable was fledging success and the fixed effect was a factor with levels “drought” and “regular” for years with and without extreme drought events, respectively. Lesser kestrel colonies were used as a random variable.

## Supplementary information

Supplementary Information.
