# Supplementary material for: Extreme events are more likely to affect the breeding success of lesser kestrels than average climate change
Source: Sci Rep. 2020 Apr 29;10:7207. doi: 10.1038/s41598-020-64087-0 (PMC7190627; doi:10.1038/s41598-020-64087-0)
Supplement: Supplementary file 1 — Supplementary Information. [file 41598_2020_64087_MOESM1_ESM.docx]

Supplementary Material for manuscript

**Extreme events are more likely to affect the breeding success of lesser kestrels than average climate change (2020)**

Marcelino, J.^a,^*, Silva, J.P.^b,c,d^, Gameiro, J.^e^, Silva, A.^f^, Rego, F.C.^a^, Moreira, F.^c,d^, Catry, I.^b,c,g^

**Author affiliations:**

^a^ Centre for Applied Ecology “Prof. Baeta Neves”/InBIO Associate Laboratory, Instituto Superior de Agronomia, Universidade de Lisboa, Tapada da Ajuda, 1349-017 Lisbon, Portugal.

^b^ CIBIO/InBIO, Centro de Investigação em Biodiversidade e Recursos Genéticos, Laboratório Associado, Universidade do Porto, Campus Agrário de Vairão, 4485–601 Vairão, Portugal

^c^ CIBIO/InBIO, Centro de Investigação em Biodiversidade e Recursos Genéticos, Instituto Superior de Agronomia, Laboratório Associado, Universidade de Lisboa, Tapada da Ajuda, 1349-017 Lisboa, Portugal

^d^ REN Biodiversity Chair, CIBIO/InBIO-UP, Centro de Investigação em Biodiversidade e Recursos Genéticos, Universidade do Porto, Campus Agrário de Vairão, Rua Padre Armando Quintas, 4485–601 Vairão, Portugal

^e^ cE3c, Centre for Ecology, Evolution and Environmental Changes, Faculdade de Ciências da Universidade de Lisboa, Campo Grande, 1749-016 Lisbon, Portugal.

^f^ Instituto Português do Mar e da Atmosfera, I.P., Rua C do Aeroporto, 1749-077, Lisboa, Portugal

^g^ School of Environmental Sciences, University of East Anglia, Norwich, UK

*Corresponding author e-mail: [joanalmarcelino@gmail.com](mailto:joanalmarcelino@gmail.com)

**This file includes:** Figures S1 to S2

**Fig.S1** Bubble plot and variogram of the spatial distribution of the model’s residuals without spatial autocorrelation structure. Colonies have equally distributed residuals in space and thus there is no evidence of spatial autocorrelation. Moran’s I statistics for the study area. Autocorrelation index is close to zero between all colony sites.

**
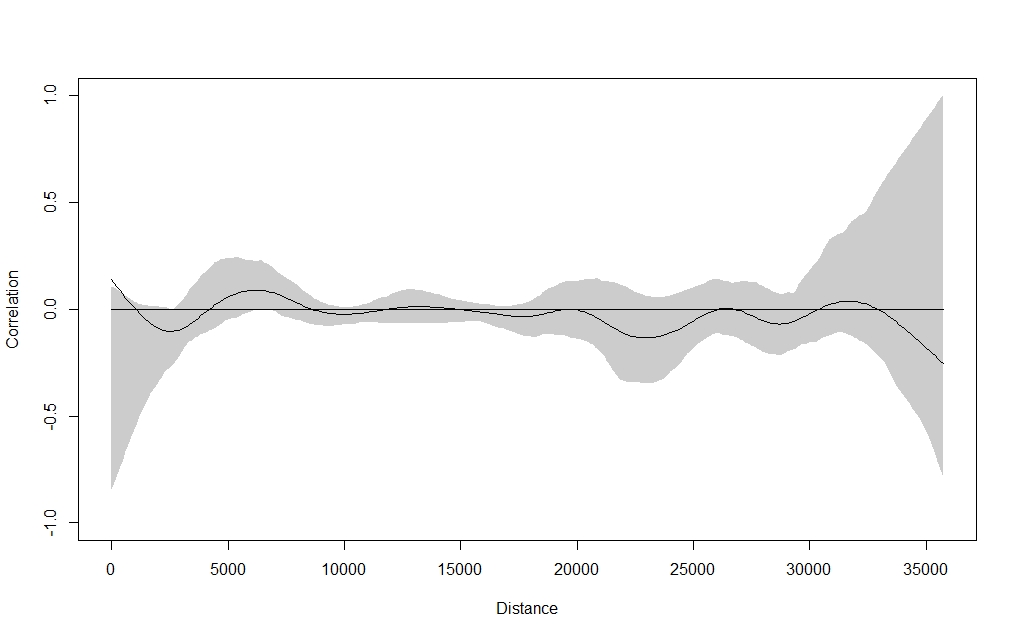

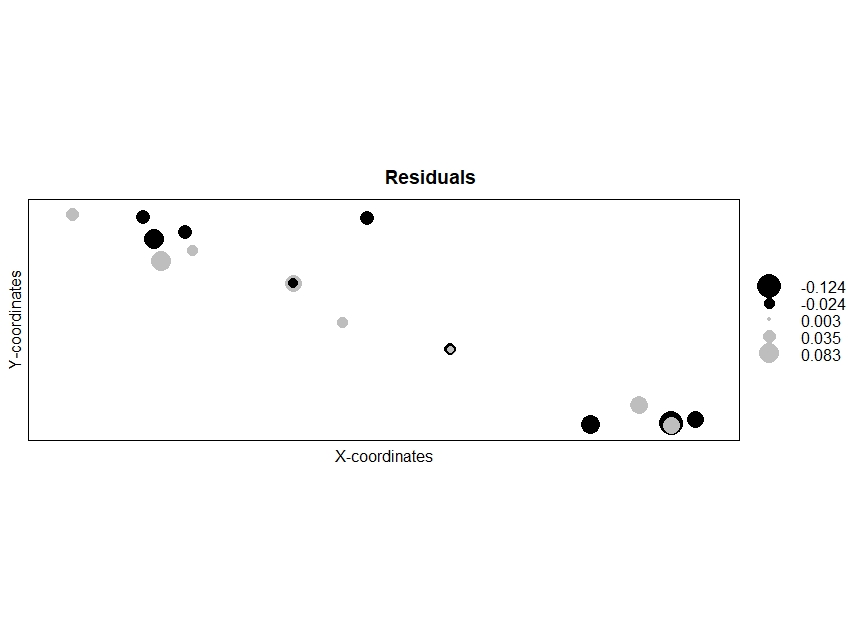
**

Moran’s I statistic = -0.04

Variance=2.5x10^-4^

p-value=0.98


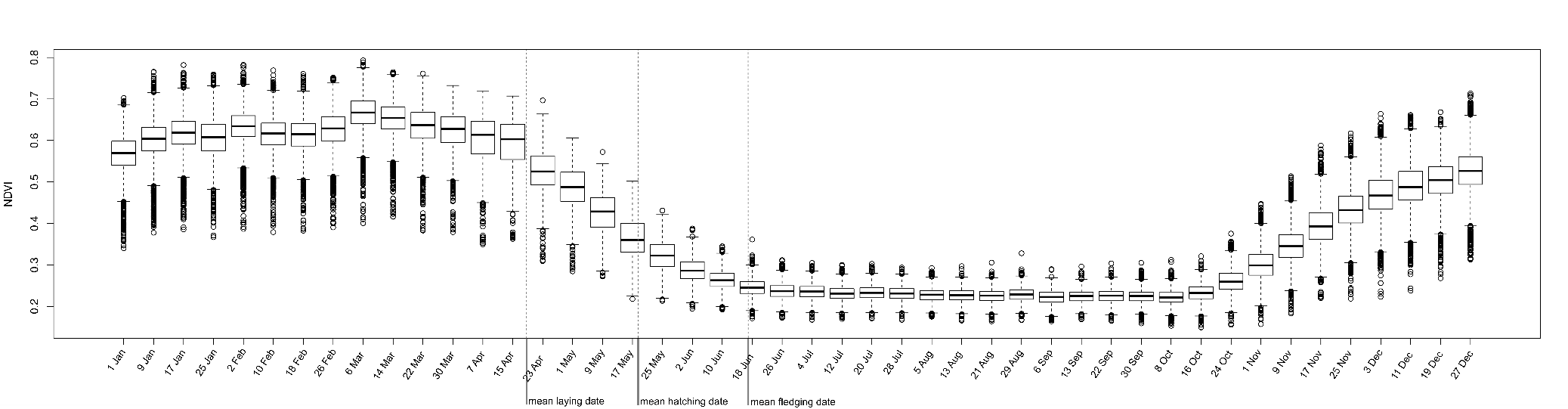


**Fig.S2** Normalized Difference Vegetation Index (NDVI) variation along a 15-year period (2003 – 2017) on an 8-day composite scale. Lesser kestrels mean laying date (28 April), mean hatching date (26 May) and mean fledging date (25 June) for the same period are shown in dashed lines. The NDVI decline clearly evident in spring months (April, May, June), when vegetation starts to dry.
